# Classification of Unmanned Aerial Vehicles Based on Acoustic Signals Obtained in External Environmental Conditions

**DOI:** 10.3390/s24175663

**Published:** 2024-08-30

**Authors:** Marzena Mięsikowska

**Affiliations:** Faculty of Mechatronics and Mechanical Engineering, Kielce University of Technology, 25-314 Kielce, Poland; marzena@tu.kielce.pl; Tel.: +48-730-930-962

**Keywords:** unmanned aerial vehicle, discriminant analysis, drone classification

## Abstract

Detection of unmanned aerial vehicles (UAVs) and their classification on the basis of acoustic signals recorded in the presence of UAVs is a very important source of information. Such information can be the basis of certain decisions. It can support the autonomy of drones and their decision-making system, enabling them to cooperate in a swarm. The aim of this study was to classify acoustic signals recorded in the presence of 17 drones while they hovered individually at a height of 8 m above the recording equipment. The signals were obtained for the drones one at a time in external environmental conditions. Mel-frequency cepstral coefficients (MFCCs) were evaluated from the recorded signals. A discriminant analysis was performed based on 12 MFCCs. The grouping factor was the drone model. The result of the classification is a score of 98.8%. This means that on the basis of acoustic signals recorded in the presence of a drone, it is possible not only to detect the object but also to classify its model.

## 1. Introduction

Drones, also known as unmanned aerial vehicles (UAVs), have become one of the most dynamically developing areas of aviation technology in recent years. Their versatility and ability to perform a variety of tasks have contributed to a wide range of applications in many fields and areas of the economy [1,2,3,4,5,6,7,8,9,10,11,12,13,14]. From agriculture and rescue operations to infrastructure inspection and transportation, drones contribute significantly to the efficiency and safety of many processes. Drones are widely used in precision agriculture [1,2]. Using drones, farmers can monitor the conditions of crops, identify farm areas that require irrigation or fertilization, and assess plant health [3]. Drones generate high-resolution images and maps, which enable optimization and efficient management of farms, thereby increasing crop yield. Drones are used to inspect hard-to-reach or dangerous locations such as high-voltage power lines, pipelines, telecommunication towers, and bridges [4,5,6,7]. In rescue operations, drones can quickly reach disaster sites, detect fires, provide first aid, and monitor situations in real time [8]. In security, drones are used for surveillance and border patrol [9,10]. Drones can be used in transport, especially medical transport [11,12,13], thus speeding up the delivery process, especially in urban areas with heavy traffic. However, to take full advantage of drones, it is necessary to effectively address issues related to autonomy, regulation, security and privacy protection [14].

Drones are equipped with a number of advanced technologies. These solutions not only increase their functionality but enable their autonomous or remotely controlled operation [15,16]. They include navigation systems for precise position tracking and flight stability, data analysis and mission planning, sensors and cameras for collecting visual and topographic data, and real-time data transmission via radio and satellite systems [17]. Artificial intelligence and machine learning can be applied to boost the autonomy of drones [18]. Machine learning, particularly deep learning, is the foundation of autonomous systems. Neural networks enable real-time analysis of large sensory data sets, allowing for object recognition, navigation in complex environments, and decision-making. The integration of sensors and the fusion of data obtained from these sensors allow for information to be obtained, inferences to be made, and specific decisions to be reached, making it possible for drones to independently map out optimal routes without obstacles [19]. Machine learning algorithms analyze power consumption patterns and adjust flight parameters to minimize battery consumption. Drones equipped with appropriate image processing and data analysis algorithms can autonomously map areas and inspect infrastructure such as bridges [20], power lines, and buildings without human intervention. Detection and classification of unmanned aerial vehicles based on acoustic signals could play a key role in the development of autonomous systems for drones.

A very important direction in the development of drones is the cooperation of drones, or the so-called “work in a swarm”. Here, it is important to avoid collision, recognize neighboring objects, detect the directions of approaching objects, achieve full autonomy of flight, and map out the path in real time. Acoustic systems can be useful for this particular application, as they can boost the autonomy of the drone. Using acoustic signals, it is possible to detect drones in natural environments even at a distance of 1 km and to indicate their exact distances, thus enhancing drone detection systems [21]. If it is possible to detect the direction of an incoming object acoustically, which is still being researched, acoustic sensors could resolve collision problems and enable drones to cooperate in a swarm. In addition, the classification of environmental signals in the presence of drones could improve their decision-making and autonomy.

When the drone is the carrier of the acoustic sensor, its noise may constitute a problem. To deal with this problem, it is necessary to discard the redundant components of the carrier from the signals and thus obtain the signals from the environment. This study carried out an acoustic analysis based on the acoustic signals obtained in the presence of various drones in order to classify the drones according to their UAV models. Following this classification, it may be possible to separate the noisy components of the drones from the useful signals. This, in turn, will make it easier to obtain signals from the environment, which will not only enable more accurate applications of drones in various fields—e.g., ecology (listening to birds), precision agriculture (acoustic observation of plantations), rescue systems, voice control of drones [22,23,24]—but will allow drones to cooperate.

Acoustic classification of UAVs can be valuable in Unmanned Ground Vehicle (UGV)-UAV cooperation in scenarios where direct communication is not possible or in environments where GNSS (Global Navigation Satellite System) signals are unavailable [25]. In such cases, the ability of UGVs to identify and interact with UAVs using acoustic signals would be a robust alternative, enhancing operational effectiveness in challenging conditions.

Despite the many advantages of drones, their use also comes with some challenges, such as regulation, security, and privacy protection [26,27,28]. The dynamic development of drone technology requires appropriate and safe legal regulations. The widespread use of drones can lead to privacy violations; thus, it requires proper regulation and protective measures. Detection of drones plays a very important role in security. A variety of sensing techniques have been proposed for drone detection, including acoustic, optical, radar detection systems and passive radiofrequency sensing [29]. Detection of small-sized drones can be very challenging [30]. Deep learning techniques, particularly the You Only Look Once (YOLO) algorithm, have been extensively explored and have shown promising results in UAV detection [31]. Privacy protection may be provided by acoustic sensors that can detect and classify objects at different heights and distances [21,32,33,34]. Acoustic systems for drone detection and classification may significantly boost security and privacy protection as well as the autonomy of drones.

The aim of this paper is to perform acoustic analysis and discriminant function analysis of acoustic signals recorded in the presence of UAVs hovering at a height of 8 m above the recording equipment in external environmental conditions. Seventeen different UAVs were used in the experiment. The acoustic analysis included the analysis of the characteristic frequencies of the background sound levels in the presence of the UAVs. Discriminant function analysis was used to investigate differences between the UAV models based on the acoustic signals recorded in the presence of each UAV. This research provides information on the classification accuracies of UAV models based on acoustic signals.

Drone detection and classification can significantly enhance security, privacy protection, and the autonomy of drones. This work investigates how acoustic signals acquired in presence of unmanned aerial vehicles can be classified. The analysis will demonstrate whether sound signals obtained in the drone regions show significant differences. The remainder of this article is organized as follows: Section 2 presents the materials and methods used in this study, the results are shown and discussed in Section 3 and Section 4, respectively, and the conclusions and future steps are presented in Section 5.

## 2. Materials and Methods

The materials and methods used in this experiment are described in the following subsections.

### 2.1. UAVs Used in the Experiment

Seventeen UAVs were used for the experiment. Their structures and models are presented in Table 1.

**Figure 1:**
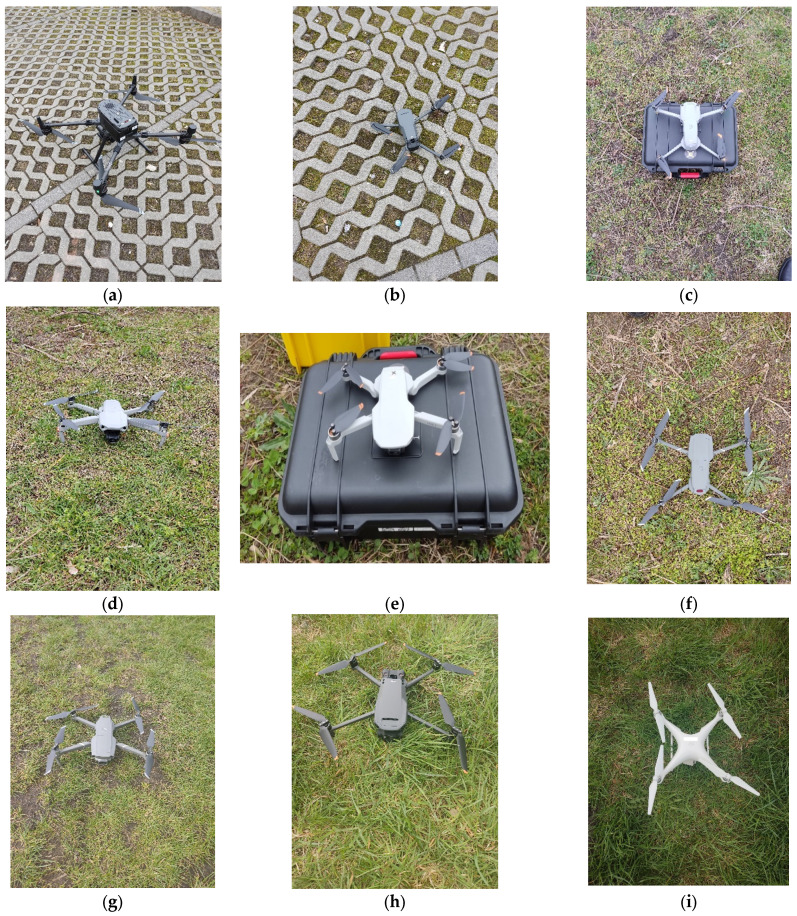

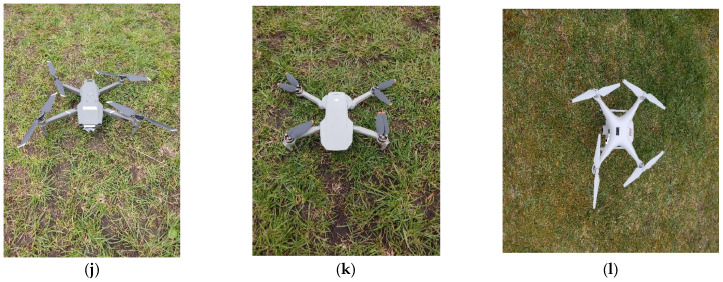
The X4 UAVs used in the experiment (**a**) D1; (**b**) D2; (**c**) D3; (**d**) D4; (**e**) D5; (**f**) D6; (**g**) D7; (**h**) D8; (**i**) D9; (**j**) D10; (**k**) D11; (**l**) D17.

**Figure 2:**
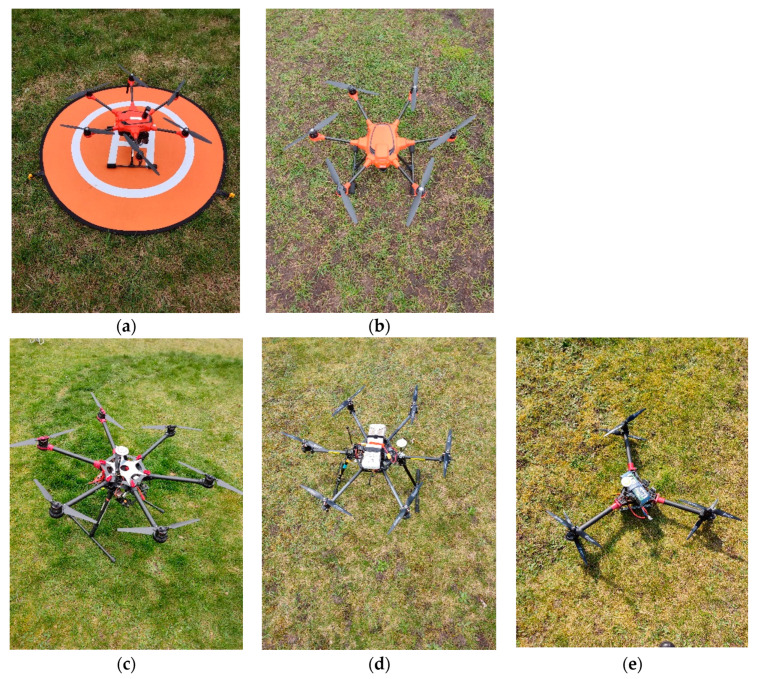
The X6 UAVs used in the experiment (**a**) D12; (**b**) D13; (**c**) D14; (**d**) D15; (**e**) D16.

Twelve of the drones used in the experiment have an X4 structure (four rotating propellers) while five have an X6 structure (six rotating propellers). Several drones of the same models were used in the experiment. Drones D5 and D11 are two different drones of the model Mavic Mini 2, drones D6 and D7 are two different drones of the model Mavic 2 Pro, drones D9 and D17 are two different drones of the model Phantom 4, and drones D2 and D8 are two different drones of the model Mavic 3. Each drone was observed separately while hovering at a height of 8 m above the recording equipment. The X4 UAVs are presented in Figure 1. The X6 UAVs are presented in Figure 2.

### 2.2. Measurement and Recording of Acoustic Signals

**Figure 3:**
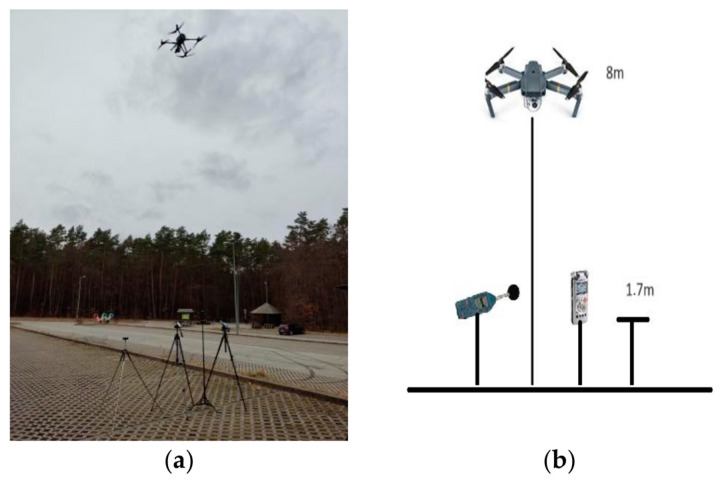
Measurement of acoustic signal. (**a**) Real environmental conditions; (**b**) illustration.

Recordings of acoustic signals in the presence of UAVs took place in four different places: in two Polish cities, Kielce and Gdańsk, and in two places in the vicinity of the city of Gdańsk. The recordings were taken separately for the seventeen UAVs. During the recording, the UAV hovered at a height of 8 m directly over the recording equipment, as shown in Figure 3.

Recordings were taken with Olympus LS-11 digital recorder and Norsonic 140 sound analyzer. The recording equipment was placed 1.7 m above the ground. For each UAV, five (5) one-minute-long recordings were taken with Olympus LS-11 at a frequency of 44.1 kHz. Five recordings were also taken for each UAV using Norsonic 140 sound analyzer.

The measurement schedule, including dates, places, weather conditions, and the UAVs recorded that day, is presented in Table 2.

### 2.3. Acoustic Analysis of Signals

Acoustic analysis of signals obtained using a Norsonic 140 sound analyzer consisted of frequency analysis of characteristic high background sound levels (peaks) and analysis of A-weighted sound levels obtained in the presence of the unmanned aerial vehicles.

### 2.4. MFCC Extraction from Recordings

Twelve Mel-Frequency Cepstral Coefficients (MFCCs) were extracted from recordings of signals in the presence of UAVs obtained with Olympus LS-11 recorder. MFCCs were used because of their efficient classification in previous experiments in which discriminant function analysis was applied to analyze sounds recorded in the presence of UAV [33]. These coefficients are also efficient in recognition systems where they provide high recognition accuracy.

### 2.5. Discriminant Analysis of MFCC

Discriminant function analysis of the 12 MFCCs was performed to investigate the differences between the UAV models. The UAV models were taken as the grouping variables and the MFCCs as the independent variables.

The discriminant analysis consisted of the discrimination stage and the classification stage. It was performed using STATISTICA software version 13.3 [36]. In the discrimination stage, the maximum number of discriminant functions evaluated was equal to the number of discriminant variables minus one. A canonical analysis was used to determine the successive functions and their canonical roots. The standardized coefficients were estimated for each discriminant function. The contribution of the variable to the discrimination between groups becomes greater as the standardized coefficients become larger. Chi-square tests with successive roots removed were investigated. The coefficient of the canonical correlation (canonical-R), which ranges between 0 (no association) and 1 (very high association), is a measure of the association between the i-canonical discriminant function and the group. Wilks’ lambda statistic, which ranges between 0 (excellent discrimination) and 1 (no discrimination), is used to determine the statistical significance of discrimination.

The classification stage followed the determination of the variables that discriminate the UAV groups. Because there were thirteen model groups, thirteen classification functions were created according to Equation (1), viz.:

K_i_(h) = c_i0_ + w_i1_mfcc_1_ + w_i2_mfcc_2_ + … + w_i12_mfcc_12_ (1)

where h is the UAV considered as a group (mavic2zoom, mavicmini2, phantom4, matrice300, mavic3, mavicair2s, mavicair2, mavic2pro, yuneech520, yuneech520ertk, s900, x6d, y6), the subscript i denotes the respective group, c_i0_ is a constant for the i-th group, w_ij_ is the weight of the j-th variable in the computation of the classification score for the i-th group, and mfcc_j_ is the observed Mel-frequency cepstral value for the respective case. The classification functions were used to determine to which group each case most likely belongs. A case was classified as belonging to the group for which it had the highest classification score, or more precisely, for which K_i_(h) assumed the highest value. The classification matrix was used to present the number of cases that were correctly classified and the number that were misclassified.

## 3. Results

The following results of the acoustic analysis and discriminant function analysis of signals detected in the presence of the UAVs in external environmental conditions were obtained in the experiment.

### 3.1. Results of Acoustic Analysis

**Figure 4:**
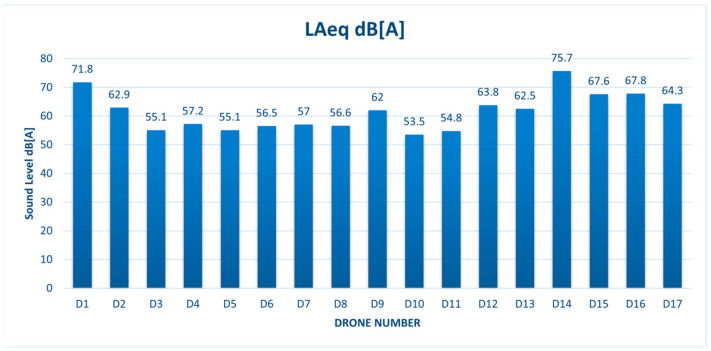
The A-weighted sound levels obtained for drones.

**Figure 5:**
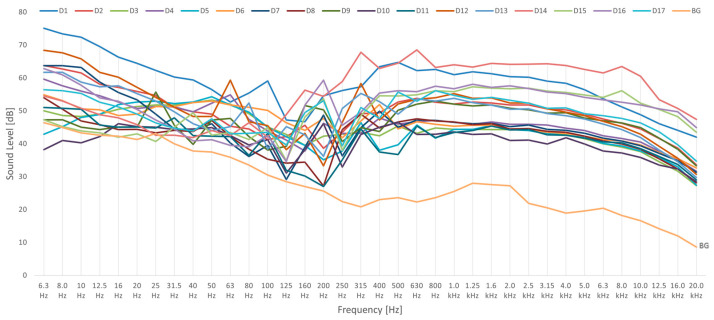
The background sound levels obtained for UAVs.

The A-weighted sound levels of the UAVs obtained with the Norsonic 140 sound analyzer in external environmental conditions are presented in Figure 4:

The background sound levels of the UAVs recorded with the Norsonic 140 sound analyzer in external environmental conditions are presented in Figure 5.

In Figure 5, the background sound levels obtained in the absence of UAVs in environmental conditions of the city of Kielce (BG) are also presented. The characteristic peak of the BG appeared at 25 Hz. The acoustic analysis showed that the X4 model resulted in smaller A-weighted and background sound levels than the X6 model. Characteristic peaks of the UAVs and their frequencies, according to Figure 5, are presented in Table 3.

According to Table 3, the presented peaks and their associated frequencies characterize the acoustic background obtained in the presence of the drones. The background sound levels are a combination of the UAV signals and the surrounding sounds. Maximum large peaks, marked in red in Table 3, show very high and easily visible peaks. Same models of drones showed similar characteristic frequencies. For example, the pair D2 and D8 showed characteristic peaks at 50 Hz, 160 Hz, 315 Hz, and 630 Hz; the pair D5 and D11 showed characteristic peaks at 315 Hz and 630 Hz; the pair D9 and D17 showed characteristic peaks at 50 Hz, 160 Hz, 200 Hz, 315 Hz, 500 Hz, and 800 Hz; and the pair D6 and D7 showed characteristic peaks at 50 Hz, 100 Hz, 200 Hz, 400 Hz, and 630 Hz. The most common characteristic frequencies for the UAVs were 50 Hz, 200 Hz, and 315 Hz.

### 3.2. Results of Discriminant Function Analysis

Discriminant function analysis was performed with 12 MFCCs as the independent variables and the UAV models as the grouping variables. The analysis showed significant main effects used in the model (Wilks’ lambda: 0.0000009; approx. F(144, 540) = 15.34; *p* < 0.00001). Eleven discriminant functions (Root0, Root1, Root2, Root3, Root4, Root5, Root6, Root7, Root8, Root9, and Root10) were created. Chi-square tests performed at the canonical stage with successive roots removed are presented in Table 4. 

According to Table 4, chi-square tests with successive roots removed were significant for all discriminant functions used in the model (R = 0.984; Wilks’ lambda = 0.0000; *p* < 0.00000). The removal of the first discriminant function resulted in a high canonical-R between groups and discriminant functions (R = 0.968). The removal of the second, third, fourth, fifth, sixth, seventh, and eight discriminant functions also resulted in a high canonical-R.

After the canonical stage and derivation of discriminant functions with 12 MFCC features that mostly discriminate between groups, the classification stage followed. The coefficients of the classification functions were determined. The classification functions were used to establish to which group each case most likely belongs. The classification matrix was obtained to show the number of cases that were correctly classified and those that were misclassified.

The coefficients of the classification functions obtained for the groups are presented in Table 5.

The results of classification of the UAV model groups using the classification functions K(h) are presented in Table 6.

The value five in Table 6 means that for five considered records of the UAV model, five were correctly classified as belonging to the considered group using the respective classification function K(h). The value 10 means that for 10 considered records of the UAV model, 10 were correctly classified. The value 9 means that for 10 considered records, 9 were correctly classified and 1 was misclassified. The value zero (0) means that no record was classified as belonging to the considered group using the function K(h). The Total row (besides the first column) contains the number of all cases classified under the given function K(h). The value 11 means that for 10 considered records of the UAV model, 10 were correctly classified and 1 record was additional and misclassified. The percentage values are the average values of correctly classified cases.

Classification of the UAV models was very accurate, as shown by the 100% value obtained for correctly classified cases, except for the Phantom 4 model, whose accuracy percentage was 90%. One record from the ten Phantom 4 drones was misclassified as a Mavic 3 model.

According to Table 6, the classification was accurate (98.8%). Discriminant analysis showed significant differences between drones of different models but no significant differences between those of the same models.

## 4. Discussion

The acoustic analysis yielded higher A-weighted sound levels and background sound levels for the X6 UAVs than for X4 UAVs. The A-weighted sound levels of the drones with an X4 structure were above 50 dB(A), while the A-weighted sound levels of the X6 drones were above 60 dB(A). The highest A-weighted sound level of 75.7 dB(A) was exhibited by the D14-X6-S900 model. The D1-X4-Matrice 300 model, also showed an A-weighted sound level above 70 dB(A) value. The background sound levels presented in Figure 5 resulted in peaks that could be characteristic for UAVs hovering at 8 m over the recording equipment, but also for other sounds in the surroundings. When investigating the BG, the surrounding factors may have a minor effect on the recordings of UAVs in this experiment. The characteristic peaks presented in Table 3 were similar for drone pairs of the same models, viz.: D2 and D8, D5 and D11, D9 and D17, and D6 and D7. The most common frequencies, which were obtained for almost all the UAV models, were 50 Hz, 200 Hz, and 315 Hz. To specify the characteristic peaks, more data records of same UAV models need to be analyzed.

Discriminant analysis based on MFCC showed significant differences between the different UAV model groups, but no significant differences between UAVs of the same models. One out of ten records of the Phantom 4 model was incorrectly classified as a Mavic 3 model, resulting in a 90% classification accuracy for the Phantom 4 group. In general, the mean classification accuracy for all of the UAV models was 98.8%. This high classification accuracy shows that UAV models can be classified based on acoustic signals. An acoustic system can serve as an additive system for other systems, e.g., vision and radar systems, to detect and classify drones. Previous research on drones shows that acoustic systems can accurately detect drones even from a distance of 1 km. Some acoustic systems can detect drones as well as the drone models. Such systems work even at night, enhancing privacy area protection. In the current study, the drones were observed and analyzed at four different places, but this had no influence on the classification accuracies.

Future research should focus on extracting features that will provide more accurate information about drones and obtaining classification scores of UAV models from other altitudes and distances. The surrounding factors may affect the accuracy of classification when increasing the distance between the UAV and recording equipment, which will be the subject of further research. Previous research on drones has shown that the acoustic signals of selected drones can be used to determine the altitudes and distances at which the drones are hovering [33]. Other information that can be obtained from drone acoustic signals may include the structure of the drone (X4 vs. X6 vs. X8 vs. X3) and its loading. Initial listening tests showed that information about the loading of a model can be obtained from the acoustic signal. The sound of the same drone with and without loading shows differences in the sound signal during listening tests. Future research should aim to numerically confirm the listening tests and obtain information about the structure of the drone and loading from acoustic signals. Such information obtained from an acoustic signal may allow us to detect an object, classify it, determine its loading, distance and height, and understand the nature of the signal. This, in turn, makes it possible to develop an acoustic sensor for an unmanned acoustic system that can perform the above activities directly from the unmanned platform. It will therefore be necessary to reject the components of the sensor carrier and acquire environmental signals. Such an operation is possible only after the nature of the drone signals has been understood, thus allowing the rejection of carrier components and the acquisition of environmental signals.

## 5. Conclusions

The aim of this study was to perform acoustic analysis and discriminant function analysis of acoustic signals recorded in the presence of UAVs hovering at a height of 8 m above the recording equipment in external environmental conditions. Seventeen different UAVs were used in the experiment.

Acoustic analysis was based on A-weighted sound levels and background sound levels in the presence of the UAVs. The acoustic analysis showed that drones of X4 model yielded smaller A-weighted and background sound levels than those of X6 model. The most common frequencies of background sound levels (peaks) obtained for almost every UAV model were 50 Hz, 200 Hz, and 315 Hz.

Discriminant function analysis showed significant differences between different UAV models, but no significant differences between the same UAV models. Classification of the UAV models was 98.8% accurate. Discriminant analysis and MFCC features showed very accurate classification results for the models.

Future research should evaluate the impact of other hovering distances of UAVs from the recording equipment on the efficiency of classification and concentrate on the classification of the structure of the drone (X4 vs. X6 vs. X8 vs. X3).

## Figures and Tables

**Table 1:** The UAVs used in the experiment.

UAV Number	UAV Structure	UAV Model
D1	X4	MATRICE 300
D2	X4	Mavic 3
D3	X4	Mavic Air 2S
D4	X4	Mavic Air 2
D5	X4	Mavic Mini 2
D6	X4	Mavic 2 Pro
D7	X4	Mavic 2 Pro
D8	X4	Mavic 3
D9	X4	Phantom 4
D10	X4	Mavic 2 Zoom
D11	X4	Mavic Mini 2
D12	X6	Yuneec H520
D13	X6	Yuneec H520E RTK
D14	X6	S900 ^1^
D15	X6	X6D ^1^
D16	X6	Y6 ^1^
D17	X4	Phantom 4

^1^ Non-commercial construction of UAV.

**Table 2:** Measurement schedule: places, dates, weather conditions, and the UAVs [35].

Day	Date	Place	Conditions	UAVs
Day 1	15 March 2023	Kielce	Temperature: 5 °C Air Pressure: 1014 hPa Humidity: 51% Wind: 22 km/h	D1, D2
Day 2	15 April 2023	Gdańsk	Temperature: 10 °C Air Pressure: 1015 hPa Humidity: 78% Wind: 25 km/h	D3, D4, D5, D6
Day 3	16 April 2023	Dębogórze, vicinity of Gdańsk	Temperature: 6 °C Air Pressure: 1022 hPa Humidity: 93% Wind: 18 km/h	D7, D8, D9
Day 4	17 April 2023	Dębogórze, vicinity of Gdańsk	Temperature: 7 °C Air Pressure: 1030 hPa Humidity: 80% Wind: 22 km/h	D10, D11, D12, D13
Day 5	18 April 2023	Łapalice, vicinity of Gdańsk	Temperature: 8 °C Air Pressure: 1033 hPa Humidity: 90% Wind: 25 km/h	D14, D15, D16, D17

**Table 3:** The characteristic frequencies of peaks (^—normal, **^**—high) of the UAVs.

UAV	D1	D2	D3	D4	D5	D6	D7	D8	D9	D10	D11	D12	D13	D14	D15	D16	D17
12.5 Hz						^											
16 Hz									^	^							
20 Hz							^	^							^		
25 Hz		^				^			**^**								
31.5 Hz				^				^			^						
40 Hz	^														**^**		
50 Hz		^	**^**		**^**	^	**^**	**^**	^	^				^		^	**^**
63 Hz				**^**					^		^	**^**			^		
80 Hz		^											**^**	^			
100 Hz	**^**					^	**^**			^	**^**	^			**^**	**^**	^
125 Hz													**^**				
160 Hz		**^**						**^**	**^**			**^**	^	**^**	^	^	^
200 Hz	^		^	**^**		**^**	**^**		^	**^**					**^**	**^**	**^**
250 Hz								^					^				
315 Hz		^	^		**^**		^	**^**	^	^	**^**	**^**	**^**	**^**			**^**
400 Hz	^			**^**		**^**	**^**								**^**		
500 Hz	^	^	**^**						^	**^**		^				^	**^**
630 Hz		^			**^**	**^**	^	^			**^**		^	**^**			
800 Hz	^		^	^					**^**						^	^	**^**
1 kHz					^					^		^	^	^			
1.25 kHz		^													^	^	
1.6 kHz				^	^	^	^	^	^	^	^			^			^
2.5 kHz		^	^		^		^	^		^					^		
4 kHz			^		^			^		^							^

**Table 4:** Chi-square tests with successive roots removed.

Roots Removed	Canonical R	Wilks’ Lambda	Chi-Square	*p*-Value
0	0.984	0.0000	995.17	0.00000
1	0.968	0.0000	749.98	0.00000
2	0.948	0.0004	551.43	0.00000
3	0.907	0.0044	387.49	0.00000
4	0.868	0.0251	263.53	0.00000
5	0.774	0.1015	163.55	0.00000
6	0.695	0.2537	98.08	0.00000
7	0.543	0.4903	50.96	0.00162
8	0.475	0.6951	26.01	0.05395
9	0.304	0.8971	7.76	0.55816
10	0.106	0.9884	0.83	0.93388

**Table 5:** The coefficients of classification functions.

ci	K(Mavic 2 Zoom)	K(Mavic Mini 2)	K(Phantom 4)	K(Matrice 300)	K(Mavic 3)	K(Mavic Air 2S)	K(Mavic Air 2)	K(Mavic 2 Pro)	K(Yuneec H520)	K(Yuneec H520E RTK)	K(S900)	K(X6D)	K(Y6)
wi1	78.68	72.36	125.46	131.42	116.42	83.64	83.56	84.21	130.48	134.29	128.93	72.83	85.57
wi2	−27.42	−52.07	−64.47	−70.11	−48.30	−45.95	−24.60	−33.13	−70.16	−69.48	−60.50	−64.23	−45.68
wi3	136.73	101.03	96.68	70.82	119.72	116.44	139.50	140.17	103.73	96.45	147.69	84.23	85.31
wi4	−140.46	−208.73	−176.03	−94.83	−150.06	−200.34	−152.54	−188.17	−183.64	−182.19	−151.99	−160.93	−139.35
wi5	188.23	246.56	279.98	253.03	247.08	234.93	187.79	209.62	303.74	303.28	321.99	185.94	208.90
wi6	−66.20	109.61	−123.52	−221.46	−151.74	38.25	−78.20	−3.50	−93.66	−107.68	−183.84	15.65	−76.86
wi7	−67.45	−112.55	−99.88	−43.51	−74.61	−103.67	−65.22	−81.71	−92.31	−103.93	−135.60	−29.11	−56.70
wi8	55.20	−20.30	125.69	122.23	94.47	45.06	58.55	44.72	133.24	153.70	118.96	55.67	58.10
wi9	−129.30	−109.27	−123.04	−135.49	−152.85	−97.02	−125.01	−162.71	−132.91	−114.35	−211.17	−77.91	−98.97
wi10	−52.32	57.16	−96.49	−147.66	−105.03	5.49	−57.61	−33.90	−100.15	−98.34	−95.28	−35.94	−44.19
wi11	304.85	340.06	359.10	268.74	372.54	318.73	314.04	367.75	337.62	324.41	409.87	129.89	197.51
wi12	−168.27	−371.09	−120.45	36.67	−66.37	−306.44	−119.78	−186.34	−208.51	−206.46	−119.42	−137.74	−74.97
ci0	−379.01	−443.49	−579.10	−496.55	−526.51	−459.50	−403.43	−466.95	−639.39	−635.76	−734.34	−379.69	−352.66

**Table 6:** The classification matrix.

Group	%	K(Mavic 2 Zoom)	K(Mavic Mini 2)	K(Phantom 4)	K(Matrice 300)	K(Mavic 3)	K(Mavic Air 2S)	K(Mavic Air 2)	K(Mavic 2 Pro)	K(Yuneec H520)	K(Yuneec H520E RTK)	K(S900)	K(X6D)	K(Y6)
Mavic 2 Zoom	100.0	5	0	0	0	0	0	0	0	0	0	0	0	0
Mavic Mini 2	100.0	0	10	0	0	0	0	0	0	0	0	0	0	0
Phantom 4	90.0	0	0	9	0	1	0	0	0	0	0	0	0	0
Matrice 300	100.0	0	0	0	5	0	0	0	0	0	0	0	0	0
Mavic 3	100.0	0	0	0	0	10	0	0	0	0	0	0	0	0
Mavic Air 2S	100.0	0	0	0	0	0	5	0	0	0	0	0	0	0
Mavic Air 2	100.0	0	0	0	0	0	0	5	0	0	0	0	0	0
Mavic 2 Pro	100.0	0	0	0	0	0	0	0	10	0	0	0	0	0
Yuneec H520	100.0	0	0	0	0	0	0	0	0	5	0	0	0	0
Yuneec H520E RTK	100.0	0	0	0	0	0	0	0	0	0	5	0	0	0
S900	100.0	0	0	0	0	0	0	0	0	0	0	5	0	0
X6D	100.0	0	0	0	0	0	0	0	0	0	0	0	5	0
Y6	100.0	0	0	0	0	0	0	0	0	0	0	0	0	5
Total	98.8	5	10	9	5	11	5	5	10	5	5	5	5	5

## Data Availability

The data presented in this study are available on the website https://t47-marzena.s3.kielce.pl/index.html accessed on 27 August 2024 under the terms and conditions of the Creative Commons Attribution (CC BY) license (https://creativecommons.org/licenses/by/4.0/ accessed on 27 August 2024). Author: Marzena Mięsikowska.
